# Full Genome Sequencing Reveals New Southern African Territories Genotypes Bringing Us Closer to Understanding True Variability of Foot-and-Mouth Disease Virus in Africa

**DOI:** 10.3390/v10040192

**Published:** 2018-04-13

**Authors:** Lidia Lasecka-Dykes, Caroline F. Wright, Antonello Di Nardo, Grace Logan, Valerie Mioulet, Terry Jackson, Tobias J. Tuthill, Nick J. Knowles, Donald P. King

**Affiliations:** The Pirbright Institute, Ash Road, Pirbright, Woking, Surrey GU24 0NF, UK; caroline.wright@pirbright.ac.uk (C.F.W.); antonello.di-nardo@pirbright.ac.uk (A.D.N.); gracie.logan@gmail.com (G.L.); valerie.mioulet@pirbright.ac.uk (V.M.); terry.jackson@pirbright.ac.uk (T.J.); toby.tuthill@pirbright.ac.uk (T.J.T.); nick.knowles@pirbright.ac.uk (N.J.K.); donald.king@pirbright.ac.uk (D.P.K.)

**Keywords:** foot-and-mouth disease (FMD), foot-and-mouth disease virus (FMDV), evolution, recombination, full-genome sequencing

## Abstract

Foot-and-mouth disease virus (FMDV) causes a highly contagious disease of cloven-hooved animals that poses a constant burden on farmers in endemic regions and threatens the livestock industries in disease-free countries. Despite the increased number of publicly available whole genome sequences, FMDV data are biased by the opportunistic nature of sampling. Since whole genomic sequences of Southern African Territories (SAT) are particularly underrepresented, this study sequenced 34 isolates from eastern and southern Africa. Phylogenetic analyses revealed two novel genotypes (that comprised 8/34 of these SAT isolates) which contained unusual 5′ untranslated and non-structural encoding regions. While recombination has occurred between these sequences, phylogeny violation analyses indicated that the high degree of sequence diversity for the novel SAT genotypes has not solely arisen from recombination events. Based on estimates of the timing of ancestral divergence, these data are interpreted as being representative of un-sampled FMDV isolates that have been subjected to geographical isolation within Africa by the effects of the Great African Rinderpest Pandemic (1887–1897), which caused a mass die-out of FMDV-susceptible hosts. These findings demonstrate that further sequencing of African FMDV isolates is likely to reveal more unusual genotypes and will allow for better understanding of natural variability and evolution of FMDV.

## 1. Introduction

Foot-and-mouth disease virus (FMDV) is the causative agent of a highly contagious and acute vesicular disease [1,2] which can lead to huge economic losses [3]. The disease is present in Africa, Asia and sporadically in South America, posing a constant threat to the global livestock industry [4].

FMDV belongs to the genus *Aphthovirus* in the family *Picornaviridae*, which are small (approximately 30 nm) positive-sense single-stranded RNA viruses with a non-enveloped icosahedral capsid. In the case of FMDV, the capsid is composed of 60 copies of each of four structural proteins VP4, VP2, VP3 and VP1, with VP1–VP3 being surface-exposed and therefore determining virus serotype [5]. The genome of FMDV is approximately 8.3 kb long and consists of a 5′ untranslated region (UTR, approximately 1.3 kb), a single open reading frame (ORF, approximately 7 kb) and a 3′ UTR (approximately 90 nucleotide long) (reviewed in [6]). Both UTRs contain conserved secondary structures that are essential for viral replication [7,8,9,10,11,12]. The beginning of the 5′ UTR contains a sequence of approximately 300 nucleotides that form a long, single stem loop known as the S-fragment which is thought to play a role in virus replication, but its exact function remains unknown [13]. Between the S-fragment and the remainder of the 5′ UTR lies a long poly(C) tract (70–250 nucleotides) of unknown function [14]. The ORF of FMDV encodes a single polyprotein which is further processed by viral proteases (L^pro^ and 3C^pro^) into partial cleavage products (i.e., precursor proteins) and 13 viral mature proteins (L^pro^, VP0, VP3, VP1, 2A, 2B, 2C, 3A, 3B1, 3B2, 3B3, 3C and 3D) [15,16,17,18]. VP0 is further cleaved into VP4 and VP2 upon encapsidation of viral RNA [19]. With the exception of L^pro^, which is a papain-like protease that also inhibits cap-dependent translation [17,20,21], all of the non-structural proteins cluster towards the 3′ end of the genome, and all directly or indirectly play a role in virus replication (reviewed in [6]).

In common with many other RNA viruses, and due to factors such as low proofreading of the viral RNA polymerase, rapid replication rate and a small genome size (reviewed in [22]), FMDV exists in samples as a population of closely related but non-identical genomic variants [23]. This inherent sequence variability is thought to enable FMDV to better explore fitness landscapes and evolve rapidly in response to host immunity and environmental pressures [24]. At the broadest scale, the high genetic diversity of FMDV is manifested by the presence of seven serotypes: O, A, C, Asia 1, and Southern African Territories (SAT) 1, SAT 2 and SAT 3 [25,26]. Additionally, across many of these serotypes, there are distinct genetic strains that circulate in different geographical regions (defining seven regional pools), each containing unique local viral topotypes and genotypes [25,26,27]. While the high viral mutation rate drives this genetic diversity, intra- and inter-serotypic recombination events can also generate genomic variability [28,29,30,31]. Mosaic structures of FMDV genomes have been observed, with the highest recombination breakpoint signals calculated to be at “the end of L^pro^-VP4” and “the end of VP1–2A” regions that may facilitate capsid switching between isolates [30,32,33,34,35].

FMDV SAT serotypes are endemic in sub-Saharan Africa where African buffalos (*Syncerus caffer*) are thought to act as a reservoir [25,26,36,37]. Maintenance of these serotypes has also been documented in domesticated livestock, such as the recent spread of SAT 2 into North Africa and the Middle East [38,39]. Serotypes A and O circulate in Africa, Asia and sporadically occur in South America; while Asia 1 isolates are normally restricted to the Asian continent [25,26]. The historical distribution of serotype C resembled that of serotypes O and A (but was less extensive) [25,26]; however, no outbreaks due to this serotype have been reported since 2004 [40].

Despite the increased number of publicly available whole genome sequences, current FMDV genomic data are biased by the opportunistic nature of sampling, with a limited characterisation of viruses circulating in Africa. This makes it difficult to evaluate the true genetic variability of FMDV, which is essential to achieve an understanding of the processes that drive the evolution of this virus. During analysis of uncharacterised African isolates, it was noticed that eight viruses from the SAT serotypes had unexpected genetic characteristics. Further phylogenetic analysis suggested that these viruses might represent “old” SAT lineages which had not been previously characterised. This study provides important insights into the potential extent of un-sampled FMDV genetic diversity which might exist within African wildlife parks.

## 2. Materials and Methods

### 2.1. Cells, Viruses and Whole Genome Sequencing

FMDV field isolates were provided by The Food and Agriculture Organization of the United Nations (FAO) World Reference Laboratory for Foot-and-Mouth Disease (WRLFMD), Pirbright. As a part of the initial diagnostic investigation for these samples, standard virus isolation protocols using tissue culture methods were adopted (i.e., 3–5 passages in primary bovine thyroid (BTy) cells or in baby hamster kidney (BHK-21) cells). Isolates were sequenced on the Illumina MiSeq (Illumina, San Diego, CA, USA) platform using a modified version of a previously described PCR-free protocol [41]. Briefly, total RNA was extracted from clarified infected cell lysates using TRIzol Reagent (Thermo Fisher Scientific, Waltham, MA, USA) as per the manufacturer’s instructions. Any residual genomic DNA was removed using DNA-free™ DNA Removal Kit (Thermo Fisher Scientific, Waltham, MA, USA) following the manufacturer’s protocol. After precipitation with 3 M sodium acetate and ethanol, 10 μL (containing from 1 pg to 5 μg) of RNA was used in a reverse transcription (RT) reaction as previously described [42] with the exception that, in addition to Random Hexamers (Bioline Reagents Ltd., London, UK), two primers that bind to conserved regions of the FMDV genome (Rev6 and NK72, previously described [41]) were included in the first incubation step and the final incubation step at 42 °C was carried out for 40 min. Second strand synthesis was carried out using the NEBNext mRNA Second Strand Synthesis Module (New England Biolabs, Ipswich, MA, USA) following the manufacturer’s protocol and subsequent cDNA extracted by the addition of an equal volume of phenol:chloroform:isoamyl alcohol (Thermo Fisher Scientific, Waltham, MA, USA) followed by 3 M sodium acetate/ethanol precipitation as described in [42]. cDNA was quantified using the Qubit dsDNA HS Assay Kit (Thermo Fisher Scientific, Waltham, MA, USA) as per the manufacturer’s instructions and a cDNA library was prepared using the Nextera XT DNA Sample Preparation Kit (Illumina, San Diego, CA, USA) following the manufacturer’s recommendations. Sequencing was carried out on the MiSeq platform using the MiSeq Reagent Kit v2 (300 cycle) chemistry (Illumina, San Diego, CA, USA).

### 2.2. Bioinformatic Analysis

#### 2.2.1. Analysis of Next Generation Sequencing Reads

The quality of subsequent FastQ files were checked using FastQC (available at http://www.bioinformatics.babraham.ac.uk/projects/fastqc/) and poor quality reads were filtered out using the Sickle algorithm [43] (available at https://github.com/najoshi/sickle). Host cell reads were removed using the FastQ Screen algorithm (available at http://www.bioinformatics.babraham.ac.uk/projects/fastq_screen/) and FMDV reads assembled de novo into contigs with IDBA-UD and using a range of kmers [44]. Only contigs which matched the FMDV library after running a Basic Local Alignment Search Tool (BLAST) algorithm [45] were assembled into consensus sequences using SeqMan Pro software implemented in the DNASTAR Lasergene 13 package (DNASTAR). To confirm the consensus, reads were mapped using Burrows-Wheeler Aligner (BWA) [46]. Mapped reads were visualised using Integrative Genomics Viewer (IGV) [47].

#### 2.2.2. RNA Structure Prediction

To predict conserved secondary RNA structures of the S-fragments from different FMDV isolates, sequences belonging to the same phylogenetic cluster were aligned using X-INS-i algorithm (which produces structure guided nucleotide alignment) implemented into MAFFT package [48,49,50]. Based on this alignment, secondary structure prediction was calculated using RNAalifold algorithm implemented in ViennaRNA Package [51,52] and structures were visualized in the Forna visualization server [53].

### 2.3. Phylogenetic Analysis

#### 2.3.1. Dataset

In order to characterize sequenced isolates, full genome sequences were compared to a subset of full genome sequences comprising SAT 1, SAT 2, SAT 3, O, A and C serotypes (available on GenBank). To reduce computational expense, as well as represent variability of serotypes O, A and C, a maximum likelihood (ML) tree was constructed using the VP1 encoding sequence extracted from all full genome sequences available from GenBank. Based on this tree, a subsample representing the highest variability for each serotype was then selected. Since, at that time, there were only 26 whole genome sequences of SAT isolates available at GenBank (SAT 1 = 11, SAT 2 = 9 and SAT 3 = 6), with exception of SAT1/NIG/1/15-SAT1/NIG/4/15 (accession no. MF678823-6) which will be characterized in our next report, all SAT isolates were included in this study. Sequences with the following GenBank accession numbers were included in this study: AY593788.1, JF749843.1, M854024.1, HQ832580.1, KM268896.1, AY593802.1, KJ608371.1, AY593751.1, AY593754.1, AY593761.1, AY593764.1, AY593766.1, KM268897.1, AJ133357.1, AJ007347.1, AY593819.1, KF112885.1, KJ206909.1, HQ632769.1, HQ632771.1, KU291242.1, KR401154.1, GU384683.1, KF694737.1, AJ539140.1, JX040491.1, KJ560291.1, DQ404170.1, KU821591.1, AY593838.1, AY593845.1, AY593844.1, JF749860.1, AY593846.1, AY593839.1, AY593842.1, AY593841.1, AY593840.1, AY593843.1, KM268899.1, AF540910.1, JX014255.1, AY593849.1, JX014256.1, AY593847.1, AY593848.1, KM268900.1, JF749862.1, KU821592.1, AY593853.1, AY593851.1, AY593850.1, KJ820999.1, KX375417.1 and KM268901.1.

#### 2.3.2. Multiple Sequence Alignment

Separate multiple sequence alignments (MSA), were prepared by dividing the FMDV genome into the S-fragment, UTRs and ORF. For the S-fragment, two alignment algorithms—E-INS-i and X-INS-i (allowing for stretches where sequences could not be aligned and guided by RNA secondary structure, respectively)—were used, both implemented in the MAFFT package [48,49]. The remainder of the 5′ UTR and 3′ UTR were aligned by global (i.e., G-INS-i) algorithm implemented in MAFFT package, while the ORF sequence was first translated using the transeq algorithm implemented in the EMBOSS package [54], then aligned using the global algorithm described above and reverse translated using the tranalign algorithm implemented in EMBOSS [54].

#### 2.3.3. Maximum Likelihood Method

Initial evolutionary history (for each genomic region) was inferred by the ML method implemented in MEGA7 software [55,56] using best nucleotide substitution model calculated by MEGA7 software and inferred based on corrected Akaike criterion (AICc) values [57,58]. Initial tree(s) for the heuristic search were obtained automatically by applying Neighbor-Joining (NJ) and BioNJ algorithms and phylogeny was tested by bootstrap method with 1000 replicates.

#### 2.3.4. Phylogenetic Signal Scan

In order to detect phylogeny violation (here used as an indicator of recombination) along the genome, whole genome MSA was subjected to TreeOrder Scan implemented in SSE v.1.3 software [59] as described previously [34] using a sliding window of 300 nucleotides with an increment of 50 nucleotides and applying the bootstrap threshold of 70% from 100 replicates for scoring of phylogeny violation. Since both SAT and Eurasian serotypes were compared together, it was difficult to identify a suitable outgroup for this analysis. Due to the difference in genome size, using bovine rhinitis B virus or equine rhinitis A virus (two other members of the *Aphthovirus* genus) as an outgroup resulted in a gapped whole genome MSA, while 50% consensus created from representatives of each phylogenetic clade appeared to be drifting towards the Eurasian clade. The TreeOrder Scan algorithm allows for analysis of unrooted trees.

#### 2.3.5. Divergence Time Estimate

Firstly, the MSA containing the ORF region was analysed in Recombination Detection Program 4 (RDP4) (i.e., RDP4 Beta 4.95 package) [60] in order to find regions of the alignment that were not a product of recombination. The largest non-recombinant region which contained the most sequences was chosen for further analysis. The dataset was assessed in TempEst v1.5.1 for the suitability for divergence time estimate [61]. The nucleotide substitution model was selected using PartitionFinder v2.1.1 [62]. The ML tree was estimated with the Randomized Axelerated Maximum Likelihood (RAxML) 8.2.11 [63] and using a bootstrap search of 1000 iterations by majority-rule tree based criteria and general time reversible substitution model with gamma distribution and invariant sites (GTR + G + I). Molecular-clock phylogeny was inferred using TreeTime 0.94 [64]. The tree was visualized and colour-coded in FigTree v1.4.3 (available at http://tree.bio.ed.ac.uk/software/figtree/).

## 3. Results and Discussion

Since whole genome sequences of SAT genotypes were particularly underrepresented in publicly available datasets, this study sequenced and characterised 34 SAT isolates collected from different locations in eastern and southern Africa, and from various host species (Table 1). We also sequenced isolates of A, O and C serotype from relevant regions which were available from the FMDV biobank held at The Pirbright Institute (Table 1).

**Figure 1:**
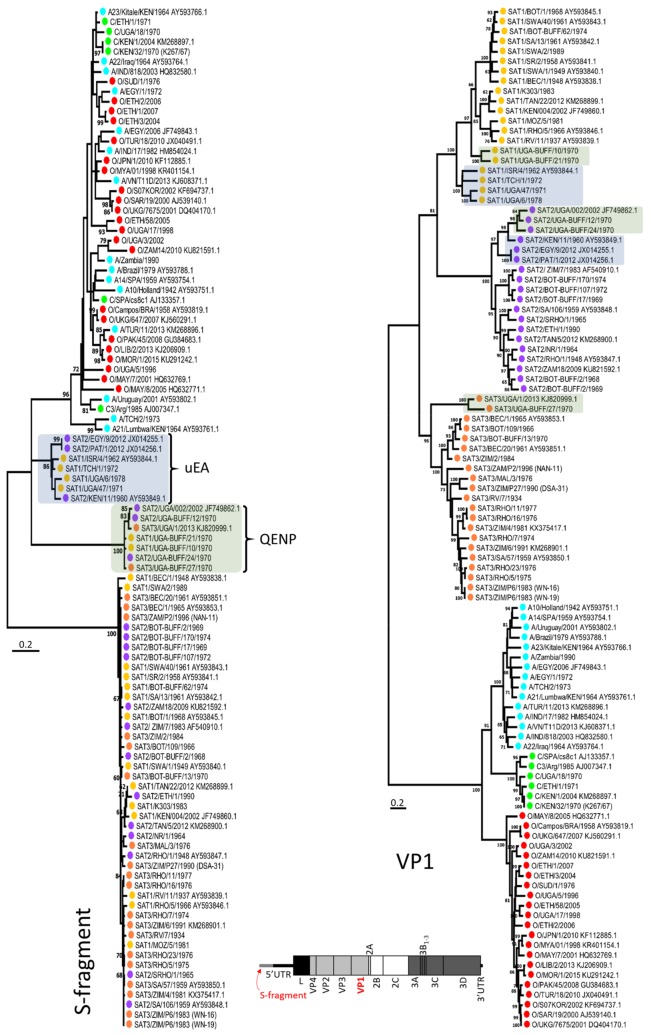
Maximum likelihood (ML) phylogenetic analysis of S-fragment sequences defining two new viral genetic clades (isolates from the Queen Elizabeth National Park (QENP) and “unusual” sequences from East Africa (uEA)). The evolutionary history based on S-fragment and VP1 sequence was inferred using the ML method and general time reversible nucleotide substitution model with gamma distribution and invariant sites (GTR + G + I) (both implemented in MEGA7 software). For each fragment the tree with the highest log likelihood is shown. The percentage of trees (with the 60% cut-off) in which the associated taxa clustered together is shown next to the branches. Initial tree for the heuristic search were obtained automatically by applying Neighbor-Joining (NJ) and BioNJ methods. Tip taxa were colour-coded according to their serotype: A—blue, C—green, O—red, Southern African Territories (SAT) 1—yellow, SAT 2—purple and SAT 3—orange. Isolates which (on the S-fragment tree) clustered in QENP and uEA clades were highlighted in green and blue, respectively. A schematic drawing of FMDV genome with highlighted in red S-fragment and VP1 encoding region is also shown. Taxa names contain full year date.

Initial analysis of these sequences revealed that 14 FMDV isolates contained atypical S-fragments (i.e., the beginning of the 5′ UTR) which were longer and difficult to align with S-fragments present in most of the other SAT and Eurasian virus genomes. Maximum likelihood (ML) phylogenetic analysis of VP1 encoding regions showed that these sequences clustered within their expected serotypes; however, when S-fragment sequences were analysed using similar approaches, these sequences formed two separate and distinct clades that contained sequences from different FMDV serotypes (Figure 1). These phylogenetic analyses adopted an MSA which contained SAT and Eurasian S-fragment sequences which shared a low nucleotide identity of approximately 47%, findings which were in common with a previous study where other SAT and non-SAT sequences were characterised [30]. Therefore, it was difficult to establish an MSA with high confidence, and this S-fragment ML tree was only used as a guide, solely to demonstrate that the newly described S-fragments sequences were distinct from other sequences and that these sequences clustered into two distinct clades (Figure 1). For the purpose of this manuscript, we named these two clades QENP (isolates from the Queen Elizabeth National Park in Uganda) and uEA (“unusual” sequences from East Africa). The QENP clade clustered SAT 1–3 isolates obtained from African buffalo and sequenced during this study (SAT 1/UGA-BUFF/10/70, SAT 1/UGA-BUFF/21/70, SAT 2/UGA-BUFF/12/70, SAT 2/UGA-BUFF/24/70 and SAT 3/UGA-BUFF/27/70) and two isolates isolated from cattle and available from GenBank (SAT 2/UGA/002/2002 and SAT 3/UGA/1/13 (accession numbers JF749862.1 and KJ820999.1, respectively)) which were previously published [65]. The uEA clade contained SAT 1–2 sequences generated in this study and isolated from cattle (SAT 1/TCH/1/72, SAT 1/UGA/47/71 and SAT 1/UGA/6/78) and four sequences available from GenBank (SAT 2/EGY/9/2012, SAT 2/KEN/11/60, SAT 1/ISR/4/62 and SAT 2/PAT/1/2012 (accession numbers JX014255.1; AY593849.1; AY593844.1 and JX014256.1, respectively) and published previously [30,38]. These sequences were generated from samples collected in Kenya, Chad, Uganda, Egypt, Palestine and Israel from different decades. The unusual phylogenetic relationships of two of these sequences (SAT 2/KEN/11/60 and SAT 1/ISR/4/62) which we define within the uEA cluster had been previously reported [30]. Further analysis showed that presented here QENP and uEA S-fragment sequences displayed 96.5% and 88.9% nucleotide identity within their clusters and 69.4% nucleotide identity between the two clusters. When comparing to the S-fragments of other SAT viruses, QENP S-fragments showed 47.9% nucleotide difference and uEA showed 41.2% nucleotide difference, while when comparing to S-fragments of Eurasian isolates, S-fragments of QENP isolates showed 44.8% nucleotide difference, while of uEA isolates showed 30.4% nucleotide difference. However once again due to lack of high confidence MSA these nucleotide distance values should be treated as a rough approximation. Similar distance scores were obtained when sequences were aligned with RNA secondary structure guided alignment algorithm and local alignment which allows for stretches of unaligned residues.

**Figure 2:**
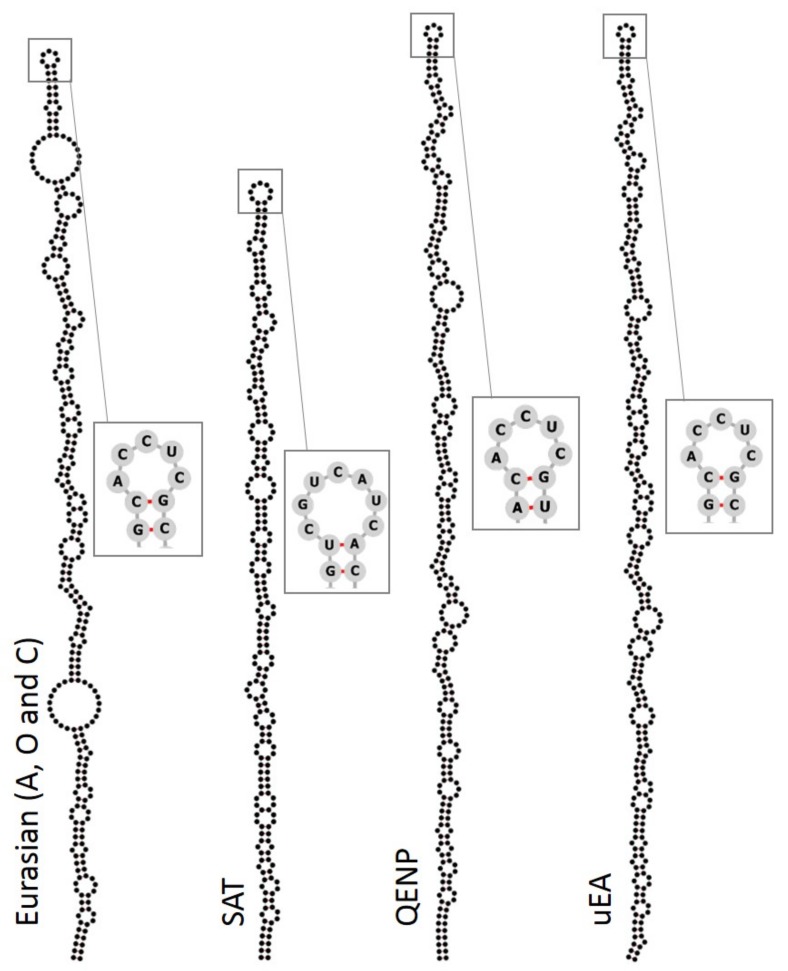
S-fragment sequences for QENP and uEA isolates can form single stem loops with similar secondary RNA structure as the S-fragments of other FMDV isolates. Separate multiple sequence alignments (MSAs) were prepared for sequences of Eurasian, SAT, QENP and uEA clusters using X-INS-i algorithm implemented in the MAFFT package. Secondary structure prediction was calculated using RNAalifold algorithm implemented in the ViennaRNA package and structures were visualized in the Forna visualization server. Sequencing of the FMDV genome often results in ambiguity at the beginning of the genome and at regions flanking the poly(C) region, therefore only regions of the S-fragment which showed unique base calling for the whole alignment were included in these RNA secondary structure predictions. Sequence at the apex of each conserved RNA secondary structure is shown, where pairing between two nucleotides is highlighted in red.

We compared the secondary RNA structures within these S-fragment sequences. Irrespective of the phylogenetic relationships, all sequences (including those for QENP and uEA clades) generated single long-stem structures (Figure 2). The QENP and uEA sequences contained a conserved ACCUC loop sequence at the apex which was shared with Eurasian isolates (Figure 2) and has been described previously [66]. The QENP and uEA S-fragment sequences are not only different in terms of the nucleotide identity, but also are longer than S-fragments of other typical SAT isolates and Eurasian isolates (Figure 2). QENP isolates had S-fragments that were 377 nucleotides long, while all the S-fragments for uEA isolates were 380–381 nucleotides long compared to 318–319 and 293–372 nucleotides long for other SAT and Eurasian isolates, respectively. Different length S-fragments have been previously characterised for other FMDV field isolates [30,31,66], and in vitro studies indicate that viruses with truncated S-fragments can still replicate [8]. Our findings add further examples of S-fragment heterogeneity that is tolerated by FMDV and motivate further studies to understand the biological role of this genomic region.

**Figure 3:**
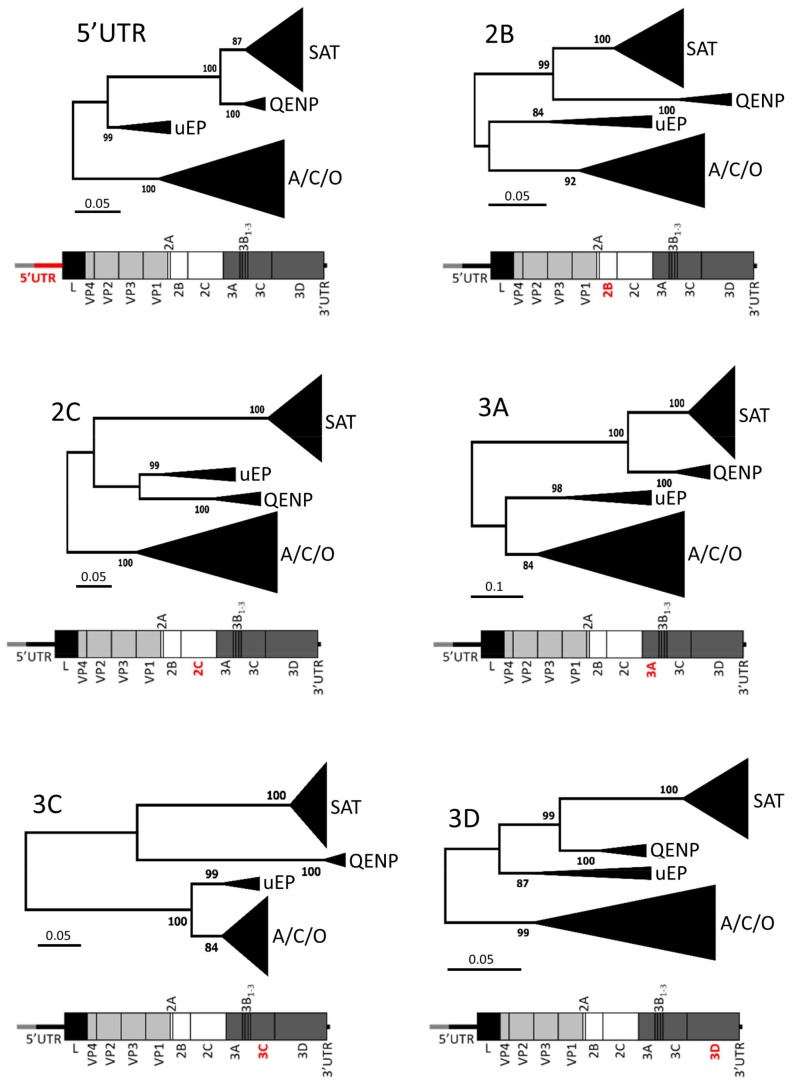
Distinct clustering for QENP and uEA isolates is displayed for different parts of the genome. The evolutionary history based on six genomic fragments (residual 5′ UTR, 2B, 2C, 3A, 3C and 3D) was inferred using the ML method and GTR + G + I nucleotide substitution model (both implemented in MEGA7 software). For each fragment, the tree with the highest log likelihood is shown. The percentage of trees (with the 60% cut-off) in which the associated taxa clustered together is shown next to the branches. Initial tree for the heuristic search were obtained automatically by applying NJ and BioNJ algorithms. A schematic drawing of the FMDV genome highlighting (in red) investigated genomic region is shown under each associated tree.

In order to investigate whether other parts of the genomes for QENP and uEA lineage isolates also generated distinct phylogenies, MSAs were prepared comprising different genomic regions encoding non-structural proteins as well as the 5′ UTR (excluding the S-fragment). While the order of the clusters changed between genomic fragments, for six genomic regions (i.e., residual 5′ UTR, 2B, 2C, 3A, 3C and 3D) viruses within the QENP and uEA clades were clustered together mirroring the tree for the S-fragment region (Figure 3). Similarly, QENP and uEA lineages formed distinct clades when ML tree was generated using full genome sequences (Figure S1). When comparing sequence diversity of the genome (excluding the S-fragment, and the variable sequence of capsid (i.e., VP2, VP3 and VP1)) the QENP sequences were 13.0% different in their nucleotide composition when comparing to other SAT viruses and 15.4% different when comparing to Eurasian isolates, while uEA were 16.6% and 13.3% different, respectively. Within the same cluster, the QENP sequences showed 95.3% nucleotide identity, and within the uEA cluster, sequences showed 91.9% nucleotide identity. Interestingly, when Dhikusooka et al. compared full genome sequence of the SAT 3/UGA/1/13 isolate to other SAT 3 full genome sequences available at the time, they concluded that these SAT 3 isolates are monophyletic [65]. Our analysis suggests that this is not the case and highlights the importance of the inclusion of sequences from other serotypes when inferring a phylogenetic relationship of non-capsid part of FMDV genome. At this moment, characterisation of SAT variability has been based on the VP1 sequence alone [67,68]; however, due to the capsid switching phenomenon [30,33,34,35], other parts of the genome should not be ignored when inferring the phylogenetic relationship of FMDV isolates.

In view of these phylogenetic relationships between different regions of the genome of the QENP and uEA sequences, we subjected the whole genome MSA to Phylogenetic Signal Scan analysis (which is part of the TreeOrder Scan algorithm implemented in SSE v.1.3 software [59]). This approach was used to detect phylogeny violation along the aligned FMDV genomes, which can be used as an indicator of recombination [34,59]. The aim of this analysis was to investigate whether QENP and uEA isolates had mosaic genome structures that might have accrued due to the accumulation of multiple recombination events (as has been previously reported for other FMDV isolates [33,34,35]), or alternatively whether these viruses represent two distinct genotypes that have generated genetic diversity independently without sequence exchange with other viruses. The TreeOrder Scan algorithm slides a user-defined window along the genome, and for each window it generates a NJ tree, each time comparing the branching order of the phylogenetic trees and calculating the number of phylogeny violations between the trees [59]. The Phylogenetic Signal Scan method of the TreeOrder Scan randomises the order of the sequences when comparing these trees in such a way that only sequences with clade support of at least 70% bootstrap cut-off remain grouped together [59]. Thus, regions of the genome without phylogenetic information are dispersed at random, while regions of the genome which contain phylogenetic information retain their space in the tree order [59]. Results were visualised in the form of a matrix of tree-order positions against the genomic regions, where large single-coloured blocks represented genomic regions with strong phylogenetic information, while regions with randomly distributed colours represented genomic regions with no or little phylogenetic information, indicative of accumulated recombination events (or other types of genomic re-assortment) [34,59].

**Figure 4:**
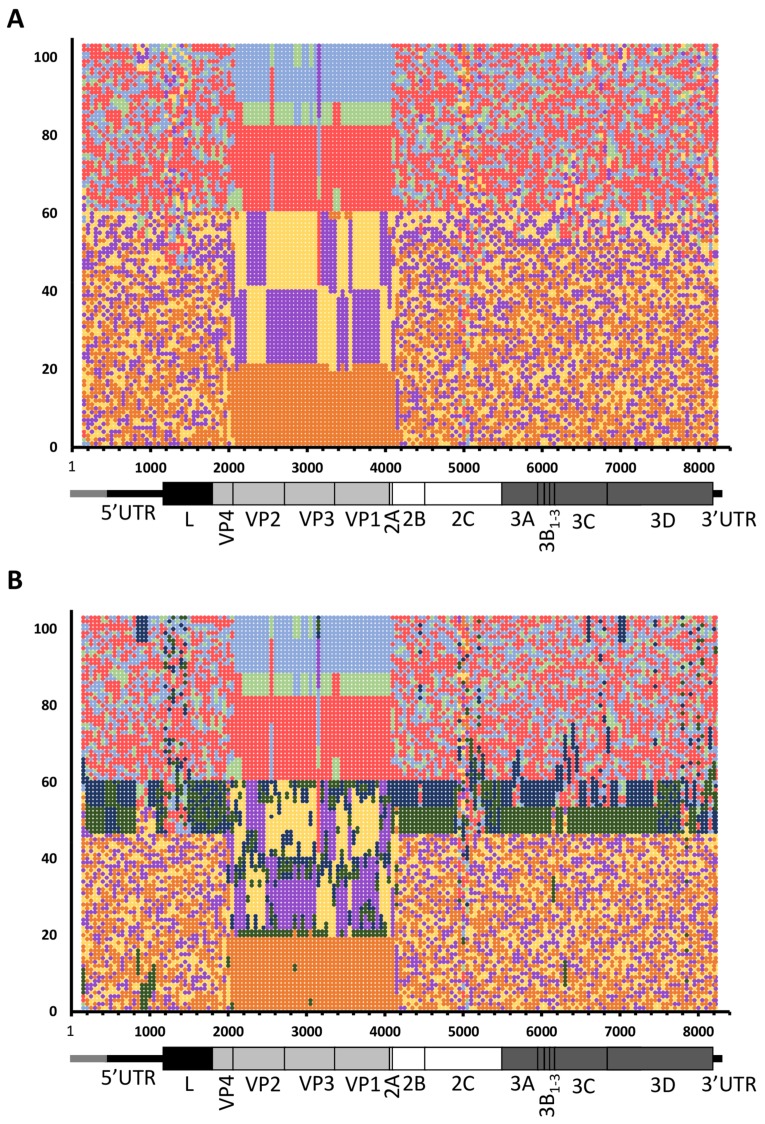
Phylogenetic Signal Scan analysis reveals the extent of distinct phylogenetic signatures of QENP and uEA FMDV lineages. (**A**) Whole genome MSA was subjected to TreeOrder Scan implemented in SSE v.1.3 software using a sliding window of 300 nucleotides with an increment of 50 nucleotides and applying a bootstrap threshold of 70% from 100 replicates for scoring of phylogeny violation. Results of the Phylogenetic Signal Scan were presented in the form of a matrix of tree-order positions against the genomic regions, where serotype A isolates are shown in light blue, serotype C in light green, serotype O in red, SAT 1 in yellow, SAT 2 in purple and SAT 3 in orange; (**B**) As in (**A**), but this time isolates from QENP and uEA clades were marked in dark green and dark blue, respectively.

In order to limit biases, we grouped all sequences according to their serotype, not specifying that we expected certain sequences to group together despite being from different serotypes (based on the results shown in Figure 1 and Figure 3, where QENP and uEA clades contained isolates from SAT 1–3 and SAT 1–2 serotypes, respectively). As expected (and as described in previous studies [33,34,35]) parts of the genome upstream and downstream of the surface exposed capsid encoding regions (i.e., VP3 to VP1) revealed a highly mosaic structure with multiple points of phylogeny violation suggestive of frequent recombination events throughout evolution of FMDV (Figure 4A). Similarly, and as expected, the Phylogenetic Signal Scan analysis revealed that while there is evidence for recombination events between different SAT isolates and different Eurasian isolates, there was relatively less evidence for recombination between SAT and Eurasian isolates (Figure 4A). The obvious interpretation of these results is that geographical separation limits the opportunities for genomic recombination between most of SAT and Eurasian FMDVs. At a finer scale, we focused analyses on sequences within the QENP and uEA clusters, where separate colours in the matrix were assigned to sequences from these isolates (Figure 4B; dark green and dark blue, respectively). These analyses revealed evidence for intra- and inter-serotypic recombination in both QENP and uEA clades, with most frequent events occurring between sequences within the same cluster (i.e., QENP isolates with other QENP isolates, and uEA isolates with other uEA isolates). These events also included inter-serotypic capsid switching. However, both QENP and uEA clusters formed distinct genomic identities with lack of mosaic structure across many parts of the genome. These findings indicate that while recombination events may have contributed to genetic diversity of these viruses, their phylogenetic relationships (as two separate clades distinct from other SAT and Eurasian viruses) cannot be solely explained by recombination events.

**Figure 5:**
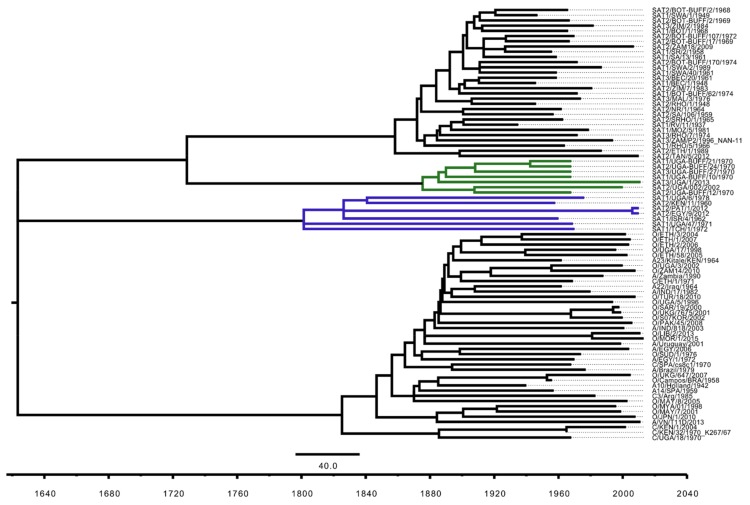
Divergence time estimates for the QENP and uEA clades. The ML tree was based on nucleotide sequence spanning through 2B and 2C encoding regions (i.e., region equivalent to 3995–4651 nucleotide position of the A/Brazil/79 (AY593788.1) sequence) and was estimated using RAxML 8.2.11 with a bootstrap search of 1000 iterations by majority-rule tree based criteria and GTR + G + I substitution model. Molecular-clock phylogeny was inferred using TreeTime 0.94 and the final tree was visualized and colour-coded in FigTree v1.4.3. Branches leading to the taxa forming QENP and uEA clades are highlighted in dark green and blue, respectively. Taxa names contain full year date.

At the end of the 19th century (1887–1897), the Great African Rinderpest Pandemic (GARP) led to a mass die-out of most of the cattle and African buffalo in sub-Saharan Africa [69], leaving only small clusters of African buffalo. This dramatic event is thought to have led to the extinction of most of the FMDV lineages that were circulating in Africa at that time [70]. It has been speculated that after the rinderpest pandemic was over, serotypes O, A and C were re-introduced into Africa from other continents due to importation of livestock into the continent [70]. We hypothesise that SAT serotype viruses re-emerged from the remaining clusters of African buffalo herds which survived the GARP. To better understand if the sequences within the QENP and uEA clade contain genomic signatures that pre-date the GARP, we tried to estimate the age of the most recent common ancestor (MRCA) of both QENP and uEA clades. In view of the highly mosaic signatures that are evident in all FMDV full genome sequences and the difficulties to accommodate recombination events in phylogenetic reconstructions, we first subjected the MSA to analysis with Recombination Detection Programme 4 (RDP4) [60] and removed from the alignment regions which were thought to originate due to recombination. While this did not necessarily eliminate all ancestral recombination signals (since RDP4 detects only unique recombination events [60]), this approach helped to compensate for recombination. This method was used to identify the longest fragment of the MSA that could be subjected to analyses using TempEst v1.5.1 package [61] for suitability for divergence time estimates. The best model was selected that described the data using PartitionFinder2 package [62]. The timed ML tree (with estimated molecular clock of 2.99 × 10^−4^ substitutions per site per year) indicated that the MRCA of QENP dated back to 1875, while the MRCA of uEA clade dated back to 1800 (Figure 5), providing evidence that MRCA of these viruses were present prior to the GARP and represent a partial picture of FMDV variability which existed in Africa prior to the GARP but which was destroyed and geographically isolated by the effects of the rinderpest pandemic.

Further sequencing of African FMDV isolates will most likely lead to the discovery of additional novel FMDV genotypes. Indeed, an unusual serotype O isolate collected from Sudan in 1960 which most likely represents O isolates which circulated in Africa prior to the GARP has been recently described [70]. However, to our knowledge, this serotype O virus represents the only example of a FMDV from O, A, or C serotypes that might have existed in Africa prior to the GARP. In contrast to the SAT serotype viruses that have remained in Africa before, throughout and since the period of the GARP, we hypothesise that it is much less likely that Eurasian serotypes (O, A or C) will contain genomic signatures representative of “old African” FMDV lineages. Therefore, in order to maximize the opportunities to uncover novel FMDV genotypes, we suggest that sequencing should focus on SAT serotype FMDVs from other isolated buffalo populations.

A better understanding of FMDV sequence space will not only help us to understand the evolutionary history of FMDV but also will infer mutational studies allowing for understanding of FMDV replication cycle (e.g., [8,71]). Defining FMDV variability also helps to understand the extent of viruses that have potential to cause FMD outbreaks in domesticated species, and several of the viruses with the novel genotypes described in this paper (such as SAT 2/EGY/9/2012 and SAT 2/PAT/1/2012) were collected from recent FMD outbreaks in North Africa and the Middle East [38,39]. Furthermore, a recent study in the QENP demonstrated the first isolation of SAT 3 virus (isolate SAT 3/UGA/1/13) from cattle which was most likely transmitted from African buffalo [65], further supporting the idea that new genotypes may emerge in domesticated species from viral reservoirs maintained by African buffalo.

## Figures and Tables

**Table 1:** Summary of foot-and-mouth disease virus (FMDV) isolates used in this study. SAT: Southern African Territories.

Serotype	Isolate Name	Year of Sampling	Country of Origin	Topotype	Host Species	GenBank Accession No.
A	EGY/1/72	1972	Egypt	AFRICA	cattle	MH053305
A	TCH/2/73	1973	Chad	AFRICA	cattle	MH053306
A	Zambia/90	1990	Zambia	AFRICA	cattle	MH053307
C	ETH/1/71	1971	Ethiopia	AFRICA	not known	MH053308
C	KEN/32/70 (K267/67)	1967	Kenya	AFRICA	cattle	MH053309
C	UGA/18/70	1970	Uganda	AFRICA	cattle	MH053310
O	ETH/3/2004	2004	Ethiopia	EA-3	cattle	MH053311
O	ETH/58/2005	2005	Ethiopia	EA-4	cattle	MH053312
O	ETH/2/2006	2006	Ethiopia	EA-3	cattle	MH053313
O	ETH/1/2007	2007	Ethiopia	EA-3	cattle	MH053314
O	SUD/1/76	1976	Sudan	WA	not known	MH053315
O	UGA/5/96	1996	Uganda	EA-1	cattle	MH053316
O	UGA/17/98	1998	Uganda	EA-4	not known	MH053317
O	UGA/3/2002	2002	Uganda	EA-2	not known	MH053318
SAT 1	BOT-BUFF/62/74	1974	Botswana	III (WZ)	*Syncerus caffer*	MH053319
SAT 1	K303/83	1983	Kenya	I (NWZ)	cattle	MH053320
SAT 1	MOZ/5/81	1981	Mozambique	II (SEZ)	cattle	MH053321
SAT 1	SWA/2/89	1989	Nambia	III (WZ)	*Syncerus caffer*	MH053322
SAT 1	TCH/1/72	1972	Chad	XI	cattle	MH053323
SAT 1	UGA/47/71	1971	Uganda	VII (EA-2)	cattle	MH053324
SAT 1	UGA/6/78	1978	Uganda	VII (EA-2)	cattle	MH053325
SAT 1	UGA-BUFF/10/70	1970	Uganda	IV (EA-1)	*Syncerus caffer*	MH053326
SAT 1	UGA-BUFF/21/70	1970	Uganda	IV (EA-1)	*Syncerus caffer*	MH053327
SAT 2	BOT-BUFF/2/68	1968	Botswana	III	*Syncerus caffer*	MH053328
SAT 2	BOT-BUFF/2/69	1969	Botswana	III	*Syncerus caffer*	MH053329
SAT 2	BOT-BUFF/17/69	1969	Botswana	II	*Syncerus caffer*	MH053330
SAT 2	BOT-BUFF/107/72	1972	Botswana	II	*Syncerus caffer*	MH053331
SAT 2	BOT-BUFF/170/74	1974	Botswana	II	*Syncerus caffer*	MH053332
SAT 2	ETH/1/90	1989	Ethiopia	IV	cattle	MH053333
SAT 2	NR/1/64	1964	Zambia (Northern Rhodesia)	III	cattle	MH053334
SAT 2	SRHO/1/65	1965	Zimbabwe (Southern Rhodesia)	I	cattle	MH053335
SAT 2	UGA-BUFF/12/70	1970	Uganda	X	*Syncerus caffer*	MH053336
SAT 2	UGA-BUFF/24/70	1970	Uganda	X	*Syncerus caffer*	MH053337
SAT 3	BOT/109/66	1966	Botswana	II (WZ)	cattle	MH053338
SAT 3	BOT-BUFF/13/70	1970	Botswana	II (WZ)	*Syncerus caffer*	MH053339
SAT 3	MAL/3/76	1976	Malawi	III (NWZ)	cattle	MH053340
SAT 3	UGA-BUFF/27/70	1970	Uganda	V	*Syncerus caffer*	MH053341
SAT 3	ZAM/P2/96 (NAN-11)	1996	Zambia	IV	*Syncerus caffer*	MH053342
SAT 3	RV/7/34	1934	Zimbabwe (Rhodesia)	VI	cattle	MH053343
SAT 3	RHO/7/74	1974	Zimbabwe (Rhodesia)	I (SEZ)	cattle	MH053344
SAT 3	RHO/5/75	1975	Zimbabwe (Rhodesia)	I (SEZ)	cattle	MH053345
SAT 3	RHO/16/76	1976	Zimbabwe (Rhodesia)	I (SEZ)	cattle	MH053346
SAT 3	RHO/23/76	1976	Zimbabwe (Rhodesia)	I (SEZ)	cattle	MH053347
SAT 3	RHO/11/77	1977	Zimbabwe (Rhodesia)	I (SEZ)	cattle	MH053348
SAT 3	ZIM/P6/83 (WN-16)	1983	Zimbabwe	I (SEZ)	*Syncerus caffer*	MH053349
SAT 3	ZIM/P6/83 (WN-19)	1983	Zimbabwe	I (SEZ)	*Syncerus caffer*	MH053350
SAT 3	ZIM/2/84	1984	Zimbabwe	II (WZ)	cattle	MH053351
SAT 3	ZIM/P27/90 (DSA-31)	1990	Zimbabwe	III (NWZ)	*Syncerus caffer*	MH053352

## Supplementary Materials

The following are available online at http://www.mdpi.com/1999-4915/10/4/192/s1.
