# Supplementary material for: Full Genome Sequencing Reveals New Southern African Territories Genotypes Bringing Us Closer to Understanding True Variability of Foot-and-Mouth Disease Virus in Africa
Source: Viruses. 2018 Apr 13;10(4):192. doi: 10.3390/v10040192 (PMC5923486; doi:10.3390/v10040192)
Supplement: Supplementary file 1 [file viruses-10-00192-s001.pdf]

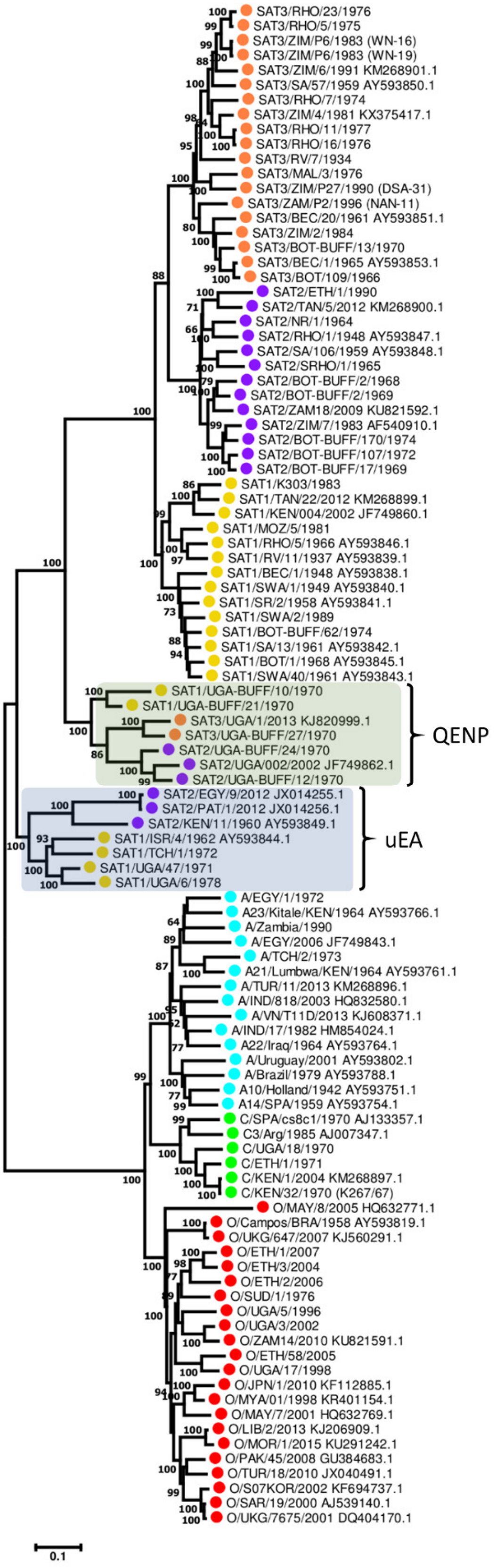

Figure S1. Maximum likelihood (ML) phylogenetic analysis of whole genome sequences confirms two new viral genetic clades (isolates from the Queen Elizabeth National Park (QENP) and "unusual" sequences from East Africa (uEA)). The evolutionary history based on the whole genomic sequences was inferred using the ML method and general time reversible nucleotide substitution model with gamma distribution and invariant sites (GTR + G + I) (both implemented in MEGA7 software). For each fragment the tree with the highest log likelihood is shown. The percentage of trees (with the 60% cut-off) in which the associated taxa clustered together is shown next to the branches. Initial tree for the heuristic search were obtained automatically by applying Neighbor-Joining (NJ) and BioNJ methods. Tip taxa were colour-coded according to their serotype: A—blue, C—green, O—red, Southern African Territories (SAT) 1—yellow, SAT 2—purple and SAT 3—orange. Isolates which clustered in QENP and uEA clades were highlighted in green and blue, respectively. Taxa names contain full year date.
